# Direct Oral Anticoagulants vs. Warfarin in Hemodialysis Patients With Atrial Fibrillation: A Systematic Review and Meta-Analysis

**DOI:** 10.3389/fcvm.2022.847286

**Published:** 2022-06-09

**Authors:** Sohil Elfar, Sara Mohamed Elzeiny, Hesham Ismail, Yahya Makkeyah, Mokhtar Ibrahim

**Affiliations:** ^1^Cardiology Department, Faculty of Medicine, Portsaid University, PortSaid, Egypt; ^2^Cardiology Department, Nasser Institute for Research and Treatment, Cairo, Egypt; ^3^Adult Intensive Care Unit, Royal Brompton and Harefield Hospitals, London, United Kingdom; ^4^Neprology Department, North West Anglia National Health Services (NHS) Foundation Trust, Huntingdon, United Kingdom; ^5^Cardiology Department, University Hospitals of Leicester, Leicester, United Kingdom

**Keywords:** hemodialysis, anticoagulants, atrial fibrillation, novel anticoagulation, renal failure, direct anticoagulant

## Abstract

**Background:**

The use of Direct Oral Anticoagulants (DOACs) in patients who have both atrial fibrillation (AF) and end-stage renal disease (ESRD) requiring hemodialysis remains controversial, with warfarin remaining the mainstay of the treatment. As hemodialysis patients were excluded from most clinical DOACs trials, the evidence of their efficacy and safety is lacking in this cohort of patients.

**Aim:**

To review the current evidence investigating safety profile and the efficacy of DOACs in comparison with warfarin in patients with AF and end-stage renal disease (ESRD) requiring hemodialysis.

**Methods and Results:**

We included five studies with a total of 34,516 patients in our meta-analysis. The outcomes were major bleeding, ischemic stroke, systemic embolization, hemorrhagic stroke, gastrointestinal bleeding, minor bleeding, and death. Of these patients, 31,472 (92.14%) received warfarin and 3,044 patients received DOACs (8.91%). No significant differences in the incidence of hemorrhagic stroke, major bleeding, hemodialysis access site bleeding, ischemic stroke, and GI bleeding were found between DOACs and warfarin. However, there were higher rates of systemic embolization, minor bleeding, and death events in patients who received DOACs than in the warfarin group (3.39% vs. 1.97%, *P*-value = 0.02), (6.78% vs. 2.2%, *P*-value 0.02), and (11.38% vs. 5.12%, *P*-value < 0.006) respectively.

**Conclusion:**

In patients on dialysis who require anticoagulation for AF, warfarin could be associated with a significant reduction in minor bleeding, systemic embolization, and death compared to DOACs. These findings need to be validated by further prospective studies to address the best strategy to deal with the increased thrombotic and bleeding risks in such patients.

## Introduction

Atrial fibrillation (AF) is the most common sustained cardiac arrhythmia in adults and is associated with an increased risk of thromboembolic stroke; therefore, anticoagulation is the cornerstone of its management (1, 2). Patients with AF who have severe chronic kidney disease (CKD) requiring dialysis have significantly higher incidence rates of ischemic stroke. In addition, there is a higher incidence of AF among patients who have end-stage renal disease (ESRD), with an increased incidence of bleeding and complications (3–5). For decades, warfarin has been the cornerstone of anticoagulation in patients with AF. However, the safety of warfarin in patients on dialysis is questioned as it may cause a higher incidence of bleeding. Additionally, the efficacy of warfarin in stroke prevention among patients with AF who are on dialysis is debatable (2, 6). Direct oral anticoagulant agents (DOACs) have been proved to have comparative efficacy and safety profiles as warfarin in reducing the risk of thromboembolic stroke and they are currently widely used in many patient groups. DOACs have been shown to be non-inferior to warfarin in mild to moderate CKD (7). However, DOACs have varying degrees of renal clearance (80% for dabigatran, 33% for rivaroxaban, and 25% for apixaban) and there is insufficient data on the safety and efficacy of DOACs in patients with stage 5 CKD (Crcl < 15 mL/min) or patients on dialysis (8). In advanced CKD (Crcl < 30 mL/min) and dialysis-dependent patients, respectively, apixaban is the most commonly used DOAC (10.4 and 10.5%), followed by rivaroxaban (9.5 and 0.8%), dabigatran (3.5 and 0.3%), and edoxaban (0.1 and 0.01%) (9). This review investigates the current evidence on the efficacy and safety profile of DOACs among patients on hemodialysis in comparison to warfarin, with stroke, systemic embolism, and major bleeding being the main points of comparison.

## Methods

### Information Sources and Search Strategy

The review protocol was registered with the international prospective register of systematic reviews (http://www.crd.york.ac.uk/PROSPERO; registration number CRD42021222346).

The following databases were searched: Cochrane Library, MEDLINE, and Google scholar database in a systematic manner from 1 August to 31 December 2020. Additionally, relevant systematic reviews were manually searched. A combination of keywords or medical terms related to hemodialysis (e.g., dialysis, ESRD), AF and anticoagulation (e.g., oral anticoagulation, DOAC, NOAC, Direct oral thrombin inhibitors, factor Xa inhibitors, dabigatran, rivaroxaban, apixaban, and Edoxaban) were used. Only studies that had human participants and were written in English were included. The research strategy is presented in the Appendix 1.

### Study Selection and Data Extraction

The search included randomized controlled trials (RCTs) and observational studies (either prospective or retrospective cohort studies). Studies with incomplete data, case reports, review articles, editorials guidelines, and duplicates were excluded.

Studies that investigated the effectiveness and safety profiles of DOACs among patients with AF and ESRD on dialysis were selected. We included the following categories of patients:

- Patients aged more than 18 years.
- Patients with ESRD on dialysis (defined as patients with a calculated glomerular filtration rate lower than 15 mL/min and requiring hemodialysis) treated with DOACs for AF.
- Patients with documented adverse outcomes (ischemic stroke, or systemic embolism, hemorrhagic stroke, major bleeding, minor bleeding, gastrointestinal bleeding, hemodialysis access site bleeding, and death).

Two authors independently performed the literature search and reviewed each title and abstract, then each of them independently reviewed the full texts of all the relevant papers. Disagreements about study eligibility were resolved via discussions among all the authors.

### Study Outcome

The primary outcomes investigated were stroke, ischemic stroke, hemorrhagic stroke, systemic embolization, major bleeding, minor bleeding, gastrointestinal (GI) bleeding, hemodialysis access site bleeding, and death.

The definition of bleeding was according to International Society on Thrombosis and Haemostasis (ISTH). Major bleeding is defined as bleeding in a critical area or organ such as intracranial, intraspinal, intraocular resulting in vision changes, retroperitoneal, intraarticular, pericardial, or intramuscular with compartment syndrome; bleeding causing a drop in hemoglobin level of 2-g/dL or more; and/or requiring transfusion of two or more units of whole blood or red cells.

Access bleeding was defined as (1) spontaneous bleeding from the arteriovenous shunt or exit site between dialysis sessions or (2) prolonged bleeding after the needles were withdrawn from the vascular access where >30 min of compression was required to achieve hemostasis.

Systemic Embolism was defined as the acute occlusion of an arterial vessel, excluding the heart, and brain.

The Preferred Reporting Items for Systematic Reviews and Meta-Analyses statement was used for this review.

### Data Extraction and Synthesis

One author extracted data from the full text of each eligible trial, then recorded the data on a specially designed Microsoft Excel data extraction form. The author responsible for extracting data was not blinded to the journal or institution.

The data extracted included type of study, number of patients, patient data regarding age, gender, CHA2DS-Vasc Score, prior stroke or embolization, heart failure, hypertension, diabetes, smoking, type of DOACs used, DOAC doses, all events, stroke, ischemic stroke, hemorrhagic stroke, systemic embolism, major bleeding (defined as fatal bleeding, bleeding at a critical site, or bleeding that required blood transfusion), minor bleeding, gastrointestinal bleeding, hemodialysis access site bleeding, and death. One author entered the data into the Cochrane Review Manager software 5.4. An independent author compared these data to the original hardcopy of data extraction forms to correct any data entry errors. If any data of interest were missing from the relevant studies, we contacted the main author or sponsor, and if these people were not reachable, the study was excluded. Two authors assessed the certainty of the evidence based on the following: perceived biases, limitations, and imprecision of the results.

The number of events and the number of patients were obtained for each trial, after which the data were combined using a fixed-effect model. For all outcomes, trial results were also combined using a random-effects model to test robustness to model choice. Relative risks and odds ratios with 95% CIs were used as summary estimates.

### Risk of Bias in Individual Studies

Two authors assessed the quality of the included studies using the Cochrane Risk-of-Bias (ROB) Methods for RCTs. For observational studies, Newcastle-Ottawa Scale was used to judge selection, comparability, and outcomes. Any disagreements between the two authors were solved *via* group discussions.

## Results

### Study Selection

**Figure 1:**
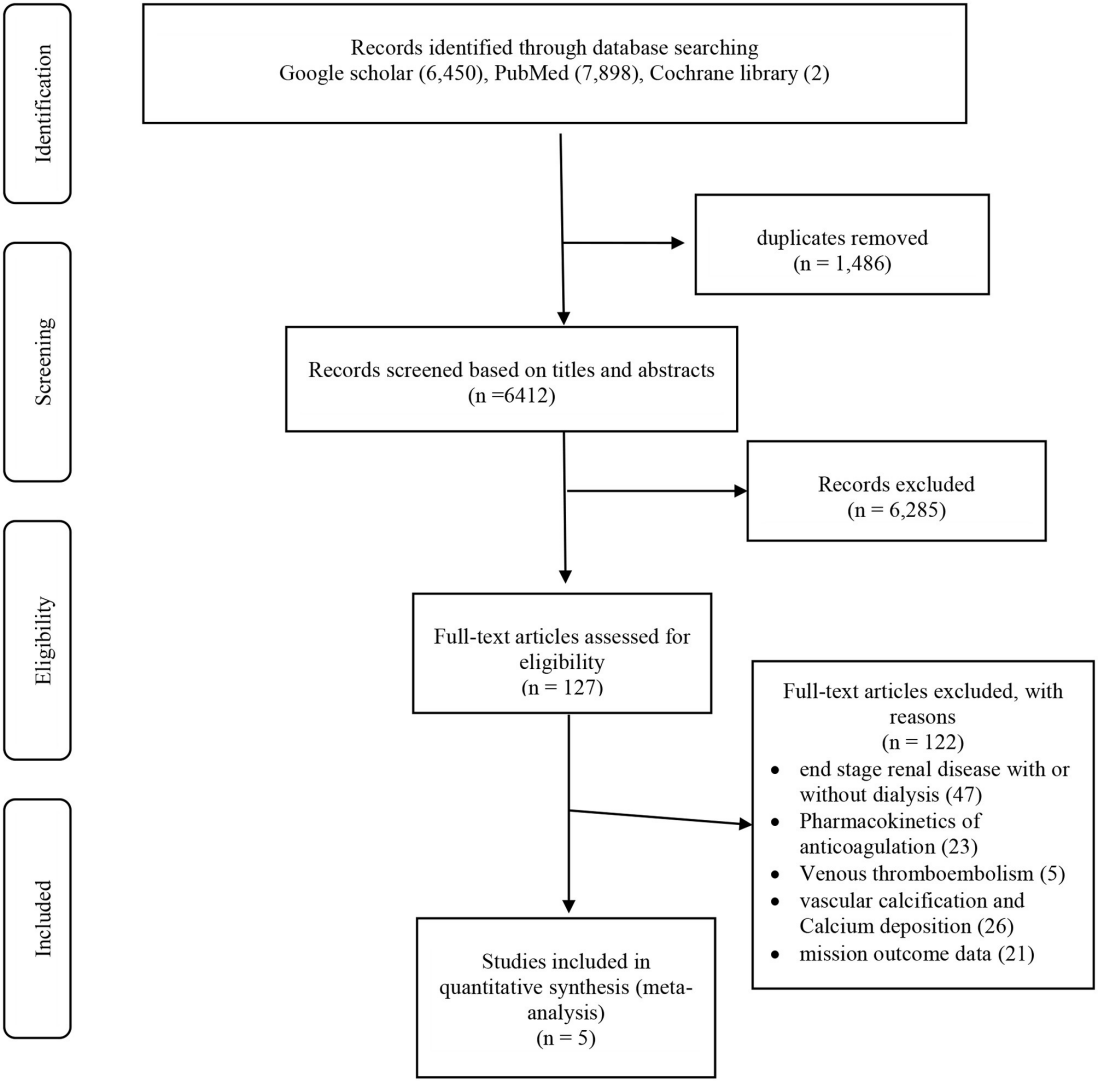
PRISMA flow diagram.

The first search of the Cochrane Library, MEDLINE, and Google scholar databases from inception to 31 December 2020, yielded 14,350 articles. After exclusion of duplicate and irrelevant items, 6,412 titles were eliminated, and 6,285 studies were excluded for being irrelevant, or were review articles, editorials, case reports, or guidelines reports. A total of 127 studies relevant to DOAC use in patients with AF on dialysis were retrieved in full text. After careful evaluation, 122 studies were excluded as 47 studies combined patients with end stage renal disease with or without dialysis, 23 studies were related to Pharmacokinetics of anticoagulation, 5 studies were on Venous thromboembolism, 26 studies were on vascular calcification and Calcium deposition, and 21 studies were having missing outcome data. Five studies were selected based on the inclusion criteria. The study selection process is presented in Figure 1.

### Study Characteristics

The selected studies were five articles including 34,516 participants with AF on dialysis. There were two RCTs, two retrospective cohort studies, and one observational prospective trial (10–14). Of these patients, 31,472 (92.14%) received warfarin, 2,473 (7.24%) received apixaban, 290 (0.85%) received rivaroxaban, and 281 (0.82%) received dabigatran. The type of included studies and basic characteristics of the patients are shown in Table 1.

**Table 1:** Study design and baseline characteristics of the included patients.

**Author name and study date**	**Study design**	**Treatment group (number of patients)**	**Age mean (SD)**	**Sex female**	**CHA2DS VASc score Mean (SD)**	**Prior stroke or embolization**	**Heart failure**	**Hypertension**	**DM**	**Smoker**
Pokorney et al. (10)	RCT	Apixaban (82)	68.75 (4.3229)	34 (41.5%)	4.0 (0.6124)	17 (20.7%)	N/A	N/A	N/A	N/A
		Warfarin (72)	67.25 (3.4611)	22 (30.6%)	4.0 (0.6124)	12 (16.7%)	N/A	N/A	N/A	N/A
Siontis et al. (11)	Retrospective cohort study	Apixaban (2,351)	68.87 (11.49)	1,071	5.27 (1.77)	778 (33.1)	1,868 (79.5)	2,342 (99.6)	1,773 (75.4)	978 (41.6)
		Warfarin (23,172)	68.15 (11.93)	10,600	5.24 (1.79)	7,683 (33.2)	17,959 (77.5)	23,079 (99.6)	17,348 (74.9)	8,819 (38.1)
Chan et al. (12)	Retrospective cohort study	Rivaroxaban (244)	66.9 (12)	96	2.2 (1.0)	14.6% (36)	14.1% (34)	84.9% (207)	67.8% (165)	N/A
		Warfarin (8,064)	70.6 (11)	3,129	2.4 (1.0)	12.0% (968)	20.8% (1,677)	88.5% (7,137)	67.9% (5,475)	N/A
		Dabigatran (281)	68.4 (12)	115	2.3 (1.0)	11.2% (31)	14.6% (41)	86.9% (244)	70.4% (198)	N/A
Sarratt et al. (13)	Retrospective, cohort study	Apixaban (40)	70.9 (5.25)	20 (50.0)	4.25 (1.4361)	6 (15.0%)	19 (47.5	33 (82.5)	22 (55.0	N/A
		Warfarin (120)	66.5 (6.75)	42(51.7)	4.75 (1.4216)	29 (24.2%)	60 (50.0)	97 (80.8)	59 (49.2)	N/A
De Vriese et al. (14)	RCT	Rivaroxaban (46)	79.525 (2.731)	11 (23.9%)	4.7 (1.4)	15 (32.6 %)	17 (37%)	N/A	20 (43.5 %)	N/A
		Warfarin (44)	79.1 (3.6894)	19 (43.13%)	4.8 (1.5)	16 (36.4%)	9(20.5%)	N/A	20 (45.5 %)	N/A

*SD, Standard deviation*.

### Quality Assessment

The Newcastle-Ottawa Scale for Observational Studies was used to assess the quality of included studies, with three studies receiving a seven-star rating (Table 2). To assess both RCTs, the Cochrane ROB tool was used and indicated a low risk of bias for both trials (Table 3).

**Table 2:** Risk of bias assessment using Newcastle-Ottawa Scale for observational studies.

			**Chan et al. (12)**	**Sarratt et al. (13)**	**Siontis et al. (11)**
Selection	Representativeness of the exposed cohort	Representative or somewhat representative of average dialysis patients in community (age/risk of stroke and bleeding)	^*^	^*^	^*^
	Selection of the non-exposed cohort	Drawn from the same community as the exposed cohort	^*^	^*^	^*^
	Ascertainment of exposure	Secure record, structured interview	^*^	^*^	^*^
	Demonstration that outcome of interest was not present at start of study	Stroke or bleeding due to anticoagulant	–	–	–
Comparability	Comparability of cohorts on the basis of the design or analysis	Study controls for renal function	^*^	^*^	^*^
		Study controls for any additional factors (history and risk of stroke and bleeding)	^*^	^*^	-
Outcome	Assessment of outcome	independent blind assessment or record linkage	^*^	^*^	^*^
	Was follow-up long enough for outcomes to occur	Follow-up > 1 year	–	–	^*^
	Adequacy of follow up of cohorts	Complete follow up (all subjects accounted for) or subjects lost to follow up unlikely to introduce bias	^*^	^*^	^*^
Score			7	7	7

* *Means equal to one point score*.

**Table 3:** Cochrane risk of bias assessment for randomized trials.

**Cochrane ROB tool for RCTs**	**Pokorney et al. (10)**	**De Vriese et al. (14)**
1. Sequence generation	Low—randomized	Low—computer-generated, web-based, locked central randomization system
2. Allocation Concealment	Low—randomized	Low—investigators (the investigator who reviewed all CT scans and the investigator who analyzed the pulse wave analysis curves) that were blinded to the treatment allocation
3. Blinding of participants and personnel	Low- open label with blinded event adjudication	Low—the primary endpoints were objectively measured by investigators that were blinded to the treatment allocation
4. Blinding of outcome assessors	Low—blind outcome assessment	Low—adjudication committee was blinded
5. Incomplete outcome data	Low	Low
6. Selective outcome reporting	Low	Low
7. Other sources of bias	Low	Low—although industry sponsored, all primary and secondary endpoints were adjudicated by blinded clinical events committee
Overall risk of bias	Low	Low

### Baseline Characteristics

**Figure 2:**
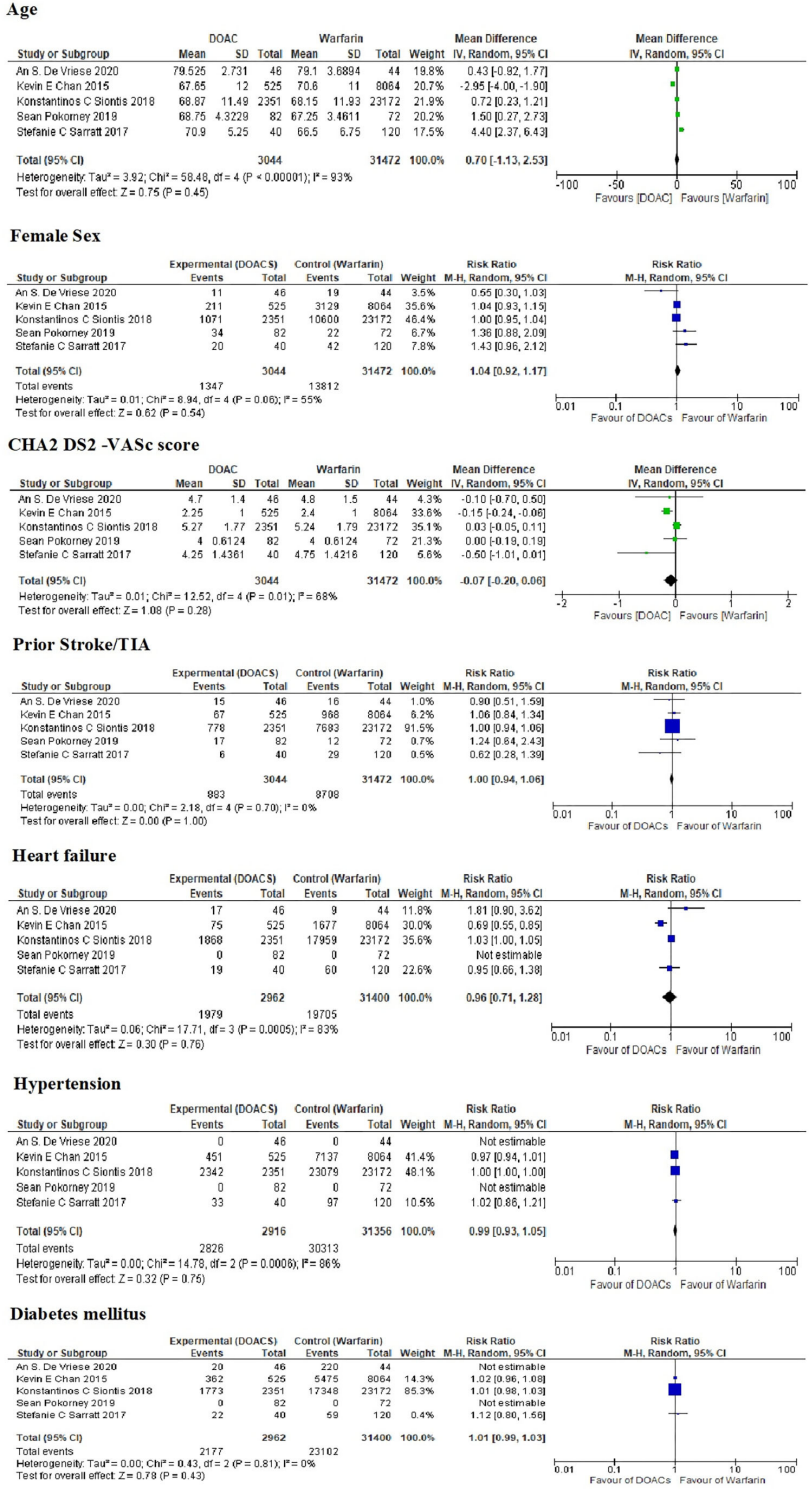
Baseline demographics and comorbidities among different studies.

Baseline demographics can be found in Table 4. The mean ages of patients in the DOACs and warfarin groups were 70.55 and 70.32 years. There was no significant difference in age between the two groups. Approximately half of the patients were females. There were no significant differences in the prevalence of comorbid conditions such as hypertension (HTN), stroke or transient ischemic attack, heart failure, and diabetes mellitus (Figure 2).

**Table 4:** Baseline demographics.

	**DOACS (*n* = 3,044)**	**WARFARIN (*n* = 31,472)**	**RR (95% CI)**	***P*** **-value**
Age mean (SD)	70.55 (4.17)	70.32 (4.6)	0.70 [−1.13, 2.53]	*P* = 0.45
Female Sex	1,347 (44.25%)	13,812 (43.88%)	1.04 [0.92, 1.17]	*P* = 0.54
CHA2 DS2 -VASc score mean (SD)	3.91 (1.35)	4.28 (1.15)	−0.07 [−0.20, 0.06]	*P* = 0.28
**Comorbid conditions (%)**				
Stroke/TIA				
*N* patients	3,044	31,472		
*N* events	883 (29%)	8708 (27.66%)	1.00 [0.94, 1.06]	*P* = 1.00
Heart failure				
*N* patients	2,962	31,400		
*N* events	1,979 (66.8%)	19,705 (62.75%)	0.96 [0.71, 1.28]	*P* = 0.76
Hypertension				
*N* patients	2,916	31,356		
*N* events	2,826 (96.9%)	30,313 (96.67%)	0.99 [0.93, 1.05]	*P* = 0.75
Diabetes mellitus				
N patients	2,962	31,400		
N events	2,177 (73.49%)	23,102 (73.57%)	1.01 [0.99, 1.03]	*P* = 0.43

*DOACS, Direct oral anticoagulants; SD, Standard deviation; TIA, transient ischemic attack; RR, risk ratio; CI, confidence interval*.

### Outcomes

**Figure 3:**
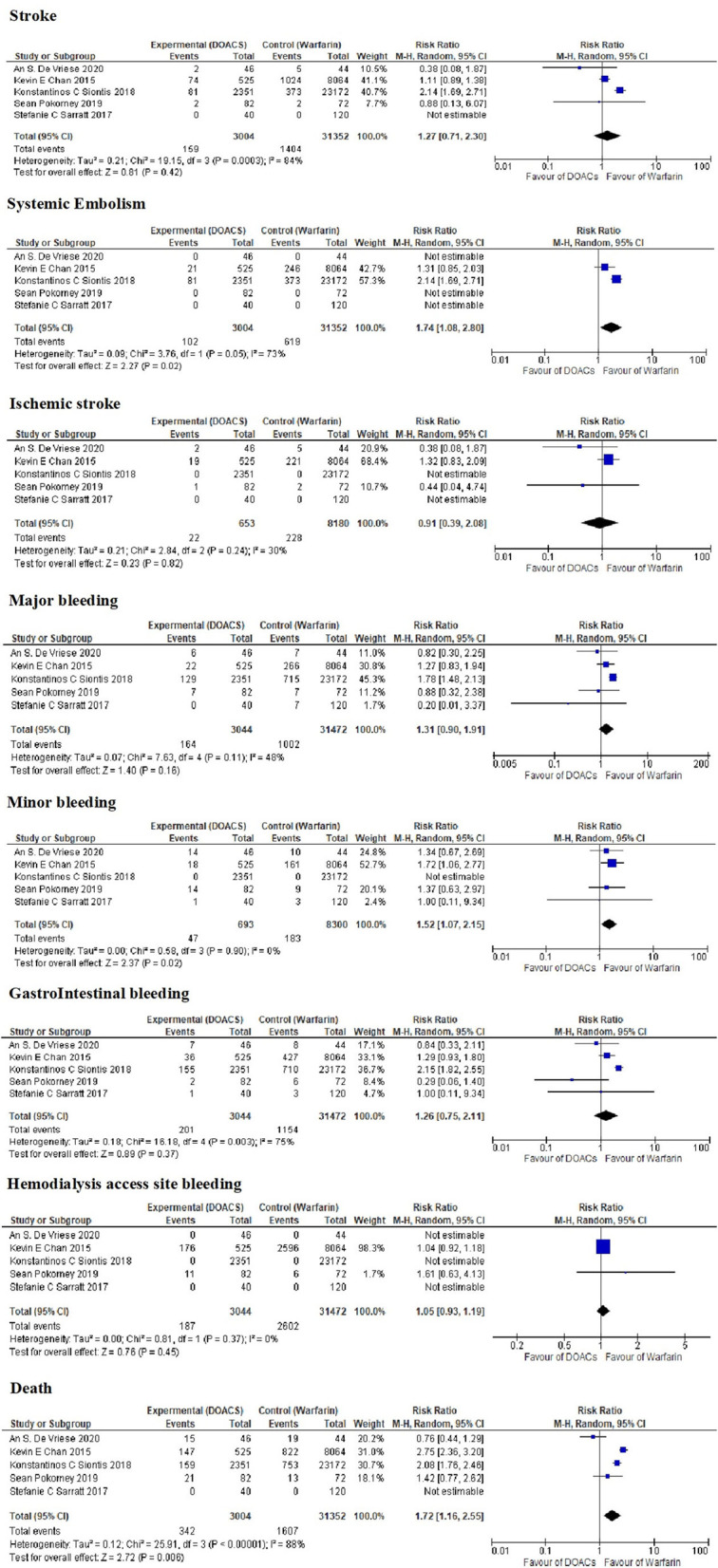
Event rates and association estimates among different studies.

The results of this study are presented in Table 5. There were no significant differences in the rates of stroke, ischemic stroke, hemorrhagic stroke, major bleeding, hemodialysis access site bleeding, and GI bleeding between patients on hemodialysis receiving DOACs and those receiving warfarin. There were higher rates of systemic embolism, minor bleeding and death in the DOACs group than warfarin group (3.39% vs. 1.97%), (6.78% vs. 2.2%), and (11.38% vs. 5.12%), respectively (Figure 3). It is important to notice that Siontis, et al. (11). described ischemic stroke and systemic emboli as one (composite) endpoint (11). It is possible that this is why the rate of systemic embolism is lower in warfarin-treated patients and why the rate of ischemic stroke does not differ significantly between treatments. We contacted the authors of the articles to obtain the respective figures; however, figures were not available. The follow up period ranged from 106 days to 540 days, two studies did not mention the follow up period (Appendix 2).

**Table 5:** Event rates and association estimates.

	**Overall (*n* = 34,516)**	**DOACS (*n* = 3,044)**	**Warfarin (*n* = 31,472)**	**RR (95% CI)**	***p*** **-value**
Stroke				
*N* patients	34,356	3,004	31,352		
*N* events	1,563 (4.54%)	159 (5.29%)	1,404 (4.47%)	1.27 [0.71, 2.30]	*P* = 0.42
Systemic Embolism				
*N* patients	34,356	3,004	31,352		
*N* events	721 (2.09%)	102 (3.39%)	619 (1.97%)	1.74 [1.08, 2.80]	*P* = 0.02
Ischemic stroke				
*N* patients	8,833	653	8,180		
*N* events	250 (2.8%)	22 (3.36%)	228 (2.78%)	0.91 [0.39, 2.08]	*P* = 0.82
Hemorrhagic stroke				
*N* patients	34,356	3,004	31,352		
*N* events	258 (0.75%)	23 (0.76%)	235 (0.74%)	0.53 [0.09, 3.25]	*P* = 0.49
Major bleeding				
*N* patients	34,516	3,044	31,472		
*N* events	1,167 (3.38%)	164 (5.38%)	1,002 (3.18%)	1.31 [0.90, 1.91]	*P* = 0.16
Minor bleeding				
*N* patients	8,993	693	8,300		
*N* events	230 (2.55%)	47 (6.78%)	183 (2.2%)	1.52 [1.07, 2.15]	*P* = 0.02
GI bleeding					
*N* patients	34,516	3,044	31,472		
*N* events	1,355 (3.92%)	201 (6.6%)	1,154 (3.66%)	1.26 [0.75, 2.11]	*P* = 0.37
Hemodialysis access site bleeding				
*N* patients	8,743	607	8136		
*N* events	2789 (31.89%)	187 (30.8%)	2602(31.9%)	1.05 [0.93, 1.19]	*P* = 0.45
Death				
*N* patients	34,352	3,004	31,352		
*N* events	1,607 (4.67%)	342 (11.38%)	1,607(5.12%)	1.72 [1.16, 2.55]	*P* < 0.006

*DOACS, direct oral anticoagulants; GI bleeding, gastrointestinal bleeding: SE, systemic embolism; RR, risk ratio; CI, confidence interval*.

## Discussion

To the best of our knowledge, this is the first meta-analysis investigating the efficacy and safety profiles of DOACs vs. warfarin in patients with AF undergoing dialysis. In contrast to the other meta-analyses that included patients at different stages of renal impairment, we focused on patients on dialysis who have been largely under-represented in previous studies.

Our study is a comprehensive review of the current evidence from five clinical trials on the use of DOACs in patients on dialysis with AF regarding safety and efficacy. It included two RCTs and three observational trials. In this systematic review, 34,516 patients with AF who were on dialysis were enrolled, 3,044 (8.9%) were DOAC users and 31,472 (81.1%) were warfarin users. The result showed that DOACs were as effective as warfarin in the prevention of stroke, hemorrhagic stroke, major bleeding, and GI bleeding. However, DOACs were associated with higher rates of systemic embolization, minor bleeding, and death events compared to warfarin.

### Stroke Risk Among Patients on Hemodialysis

In their meta-analysis, Zimmerman et al. demonstrated that 11.6% of patients on hemodialysis had AF. They also reported that the annual incidence of stroke in patients with AF on dialysis was 5.2% as opposed to 1.9% in those without AF (5). Other studies have challenged this idea and showed that AF is not an independent risk factor for stroke (15). Potential explanations are the high competing risk of mortality, a protective effect of heparin administration during dialysis, and the high prevalence of subclinical AF in patients on dialysis contaminating the “no AF” cohort in observational studies (16). Despite the paucity of studies on anticoagulation therapy in patients on dialysis, guidelines still adopt formal anticoagulation therapy for patients with high thrombotic risks. In fact, these patients were either excluded or under-represented in most of the DOAC trials (17–19).

The risk of death is higher in ESRD patients with AF than in those without AF. It is worth noting that the incidence and prevalence of AF in patients on dialysis appear to be higher because of increasing age, higher prevalence of other comorbidities, increased attention, and more people “looking for AF” with different devices e.g., 12-Lead ECG, pulse palpation, smartwatch, implantable loop recorder, ambulatory patch ECG, and multi-lead Holter monitor (5, 20–23).

### Use of Warfarin in Patients on Hemodialysis

Warfarin is the most frequently used drug for anticoagulation in AF. Nonetheless, the risk of bleeding in patients on dialysis is increased with warfarin, which may be caused by platelet dysfunction. Platelet dysfunction occurs both as a result of intrinsic platelet abnormalities and impaired platelet-vessel wall interaction. The classic stages of platelet response to injury (activation, recruitment, adhesion, and aggregation) are all defective in patients with renal failure. Although dialysis may partially overcome these defects, it cannot totally correct them. The dialysis process itself may, in fact, contribute to bleeding. Hemodialysis is also associated with thrombosis as a result of chronic platelet activation due to contact with artificial surfaces during dialysis (24).

In our meta-analysis, four out of the five papers reported a target INR of 2–3, and one study (14) reported a mean INR of 3.5. Lower doses of warfarin are sometimes preferred in patients on dialysis to achieve a lower INR target because of the increased risk of bleeding. However standard dosing has been shown to be superior in stroke prevention without increased bleeding risk (19, 24–26).

The use of warfarin did not bring about a significant reduction in the rates of stroke and death and was associated with an increased risk of major bleeding as reported by previous meta-analyses (27). Warfarin is thought to accelerate vascular calcification and aortic stenosis, which might increase the risk of ischemic stroke (2, 28). Additionally, the use of warfarin was associated with a higher risk of anticoagulant-induced renal injury than the use of DOACs (29, 30).

### Use of DOACs in Patients on Dialysis

The Renal-AF trial recently investigated the use of DOACs in patients on dialysis. In this study, 154 patients with AF on dialysis were randomly assigned to either the apixaban 5 mg BID (*N* = 82) or warfarin (*N* = 72) groups, with a target INR of 2–3 and time in therapeutic range (TTR) for warfarin of 44.3%. They included patients with AF who were on hemodialysis, had CHA2DS2-VASc scores of ≥2, and were candidates for OAC and excluded patients with moderate to severe mitral stenosis, patients who needed aspirin at doses of >81 mg, patients who needed dual antiplatelet therapy, patients with indications for OAC other than AF, and patients with life expectancies of <3 months. The follow-up period was 1 year. The results showed that apixaban 5 mg BID caused similar rates of major bleeding (8.5%) as warfarin (9.7%) and clinically relevant non-major bleeding (31.5%) as warfarin (25.5%). Also, there was no significant difference in the incidence of stroke between the two groups (2.4% vs. 2.8%). It is important to note that the trial was stopped earlier than planned due to the lack of funding and the fact that a majority of the patients on warfarin were in the subtherapeutic range with TTR (44.3%) (10).

Similarly, Sarratt, et al. (13) compared the rates of major bleeding, clinically relevant non-major bleeding, and minor bleeding between apixaban and warfarin in patients with AF on hemodialysis. Theirs was a single-center retrospective cohort study. They found no significant differences between the two groups (13).

Siontis, et al. (11) published the results of their large, retrospective cohort study that included 25,523 patients from the United States Renal Data System (October 2010 to December 2015). According to the results of this study, standard-dose apixaban (5 mg BID) was associated with significantly lower rates of stroke, systemic embolism, and death compared to either warfarin or low-dose apixaban (2.5 mg BID). In addition, apixaban, irrespective of the dose (5 mg bd or 2.5 mg bd), was associated with lower rates of major bleeding events than warfarin. The standard dose was associated with lower rates of thromboembolic events and death. These data support the growing evidence that recommends the safety profile of apixaban in this high-risk patient group and warrants further randomized clinical trials to further confirm the results of earlier studies (11).

Regarding rivaroxaban and other DOACs, there was some discrepancy in results between different studies. Chan, et al. (12) used Poisson regression analysis to compare rivaroxaban and dabigatran to warfarin in patients with ESRD. Although the exact figures could not be obtained and were not included in our statistical analysis, the study concluded that dabigatran and rivaroxaban were associated with higher risks of hospitalization and hemorrhagic death compared to warfarin. On the contrary, De Vriese, et al. (14) investigated the topic from a different point of view. They assessed the relationship between vitamin K status and the risk of bleeding in patients with ESRD, with the hypothesis that warfarin could cause functional vitamin K deficiency, which might lead to more bleeding and the acceleration of vascular calcification, which was assessed by CT calcium scores in the major vessels. Patients with non-valvular AF and CHA2DS2-VASc scores of ≥2 were randomly divided into 3 groups: warfarin with INR 2–3, rivaroxaban 10 mg OD, and rivaroxaban 10 mg OD with a vitamin K supplement. The results showed that rivaroxaban was associated with lower rates of life-threatening and major bleeding events compared to warfarin; however, no significant differences in calcium scores were noted.

### Difference in Clinical Outcome Between DOACs and Warfarin

Our data showed that DOACs are as effective as warfarin in the prevention of stroke, hemorrhagic stroke, major bleeding, and GI bleeding.

Despite the fact that each individual study did not find a significant difference in the rates of minor bleeding, systemic embolization, and mortality, the pooled data of the five studies showed a significant increase in the mortality rate among patients that took DOACs compared to patients that took warfarin [10.1% and 5.1% (*p* < 0.001)]. This difference can be attributed to several factors.

Firstly, there was a large impact of two big observational studies (more than 85% of patients) with the inherited bias to non-randomized assignments of the observational studies. Looking at the individual studies that reported this difference in mortality, Chan's study included both dabigatran and rivaroxaban at full and reduced doses (12), while Siontis, et al. (11) used both doses of apixaban. The first study included a DOAC that is clearly not suitable in ESRD–i.e., dabigatran. This drug reported renal clearance values of up to 85%, second compartment pharmacokinetics, low protein binding (thus dialyzable and prone to large variations in plasma concentrations), and a very clear-cut dose relationship with respect to thrombosis/bleeding shown in a large sub-study of RELY including more than 9,000 patients (31). Secondly, In the Siontis study, patients had high mean CHA2DS2- VASc scores of up to 5.2 ± 1.8, unlike other studies reflecting multiple comorbidities (10). Thirdly, most patients on warfarin in Chan's study were sub-therapeutic (only 13.7% of patients had ≥60% of their INR readings within the target of 2–3) (12).

There were also significant differences in the rates of minor bleeding and systemic embolization, with the lower rates occurring in the warfarin arm. The possible explanations for this difference include: the use of reduced DOAC doses in some patients, the inability to monitor the efficacy of anticoagulation, and the variable clearance of DOACs with hemodialysis.

The doses of DOACs varied between studies; De Vriese's group used a reduced dose of rivaroxaban 10 mg while the other studies combined reduced doses of DOACs. We contacted the authors to verify if separate data were available for both doses but unfortunately, this was not the case.

One of the major advantages of DOACs over warfarin is that there is no need for laboratory monitoring. However, in certain patient cohorts, including patients on dialysis, it might be important to ascertain either the actual DOAC concentration (quantitative) or the effect of DOACs (qualitative). None of the included studies assessed the level or the effect of DOACs, which may reflect the real-world situation with DOACs monitoring.

Unlike apixaban and edoxaban that are cleared by dialysis in 6 and 9%, respectively, dabigatran is cleared up 50%−60% within 4 h of hemodialysis. There were no published data on rivaroxaban clearance by dialysis. This reflects why apixaban was used the most in our study groups (32).

The fact that there is no need for routine laboratory monitoring of the effects of DOACs can lead to either undertreatment or overtreatment, which might be another reason for the significant differences in some parameters. Our study highlights the potential role of monitoring the level and effect of DOACs in this cohort of patients.

### Ongoing Trials to Study Stroke Prevention in Patients With AF on Dialysis

There are three upcoming trials that would further depict the role of oral anticoagulation in patients with ESRD on dialysis and help establish the optimal pharmacological or interventional strategy (left atrial appendage occlusion) in this population.

The German AF network also registered an open-label RCT (AXADIA), recruiting patients since April 2017. This trial will end in July 2023. The AXADIA trial will assess the safety of apixaban vs. phenprocoumon in patients with AF on hemodialysis (33).

The AVKDIAL trial is comparing the hemorrhagic and thrombotic risks of oral anticoagulation with that of no anticoagulation in hemodialyzed patients with AF. The target INR (2–3) is monitored at least once per week (34).

The SAFE-D trial (ClinicalTrials.gov Identifier: NCT03987711) is an open-label randomized trial involving patients with ESRD and AF on dialysis to compare three arms: apixaban (both 5 mg and 2.5 mg twice daily), warfarin, and no anticoagulation, for 26 weeks (35).

## Limitations

Our study has some limitations that warrant consideration. Firstly, there were only five studies that met the inclusion criteria in our meta-analysis with a relatively small number (3,044) of patients on DOACs.

Secondly, we acknowledge the heterogeneity of the five included studies. These studies have different study designs, with two being randomized control trials while the other three were observational studies that come with an inherent selection bias. It is important to notice that the difference in mortality was attributed to two large observational studies.

Additionally, different DOAC drugs with different doses were used. Furthermore, the studies included had heterogeneous inclusion/exclusion criteria and varying definitions of each outcome and follow-up duration.

Similar to other meta-analyses, the endpoint definition may vary between studies on safety and efficacy outcomes. Some studies did not clearly define the stroke subtypes, systemic embolism, and bleeding subtypes (major or minor). Additionally, they did not clarify the etiology of bleeding endpoints, especially cerebral hemorrhage.

Finally, there were some patients receiving antiplatelet therapy who could not accurately be identified in the retrospective studies but could have possibly affected our results.

## Conclusion

This meta-analysis has demonstrated that in patients on dialysis who need anticoagulation for AF, warfarin could be associated with a significant reduction in the rates of minor bleeding, systemic embolization, and death compared to DOACs. These findings need to be validated by further prospective studies to address the best strategy to deal with the increased thrombotic and bleeding risks in such patients.

## Data Availability Statement

The original contributions presented in the study are included in the article/Supplementary Material, further inquiries can be directed to the corresponding author.

## Author Contributions

All authors listed have made a substantial, direct, and intellectual contribution to the work and approved it for publication.

## Conflict of Interest

The authors declare that the research was conducted in the absence of any commercial or financial relationships that could be construed as a potential conflict of interest.

## Publisher's Note

All claims expressed in this article are solely those of the authors and do not necessarily represent those of their affiliated organizations, or those of the publisher, the editors and the reviewers. Any product that may be evaluated in this article, or claim that may be made by its manufacturer, is not guaranteed or endorsed by the publisher.
