# Supplementary material for: Direct Oral Anticoagulants vs. Warfarin in Hemodialysis Patients With Atrial Fibrillation: A Systematic Review and Meta-Analysis
Source: Front Cardiovasc Med. 2022 Jun 9;9:847286. doi: 10.3389/fcvm.2022.847286 (PMC9218480; doi:10.3389/fcvm.2022.847286)
Supplement: Supplementary file 1 [file Data_Sheet_1.docx]

## Appendix 1 containing the research strategy

.

**Appendix 1.1 CENTRAL (The Cochrane Library)**

ID Search Hits

#1 MeSH descriptor: [Factor Xa Inhibitors] explode all trees 553

#2 MeSH descriptor: [Atrial Fibrillation] explode all trees 4665

#3 MeSH descriptor: [Dabigatran] explode all trees 311

#4 MeSH descriptor: [Rivaroxaban] explode all trees 529

#5 edoxaban 590

#6 apixaban 963

#7 rivaroxaban 1713

#8 dabigatran 1078

#9 #1 OR #3 OR #4 OR #5 OR #6 OR #7 OR #8 3603

#10 #2 AND #9 497

#11 MeSH descriptor: [Renal Dialysis] explode all trees 5132

#12 #10 AND #11 2

**Appendix 1.2 Google Scholar**

“Atrial Fibrillation” and “dabigatran” OR “rivaroxaban” OR “apixaban” OR “factor Xa inhibitor” OR “NOAC” OR “DOAC” AND "dialysis"

**Appendix 1.3 PubMed**

((((((("atrial fibrillation"[MeSH Terms] OR ("atrial fibrillation"[MeSH Terms] OR ("atrial"[All Fields] AND "fibrillation"[All Fields]) OR "atrial fibrillation"[All Fields]) OR "atrial fibrillation"[MeSH Terms] OR ("atrial fibrillation"[MeSH Terms] OR ("atrial"[All Fields] AND "fibrillation"[All Fields]) OR "atrial fibrillation"[All Fields] OR ("atrial"[All Fields] AND "fibrillations"[All Fields]) OR "atrial fibrillations"[All Fields])) AND "new"[All Fields] AND ("mouth"[MeSH Terms] OR "mouth"[All Fields] OR "oral"[All Fields])) OR ("mouth"[MeSH Terms] OR "mouth"[All Fields] OR "mouths"[All Fields] OR "mouth s"[All Fields] OR "mouthed"[All Fields] OR "mouthful"[All Fields] OR "mouthfuls"[All Fields] OR "mouthing"[All Fields]) OR "mouth"[MeSH Terms]) AND "anticoagulants"[MeSH Terms]) OR ("anticoagulants"[Pharmacological Action] OR "anticoagulants"[MeSH Terms] OR "anticoagulants"[All Fields] OR "anticoagulant"[All Fields] OR "anticoagulate"[All Fields] OR "anticoagulated"[All Fields] OR "anticoagulating"[All Fields] OR "anticoagulation"[All Fields] OR "anticoagulations"[All Fields] OR "anticoagulative"[All Fields]) OR "DOAC"[All Fields] OR ("n 4 oleylcytosine arabinoside"[Supplementary Concept] OR "n 4 oleylcytosine arabinoside"[All Fields] OR "noac"[All Fields]) OR ("factor xa inhibitors"[Pharmacological Action] OR "factor xa inhibitors"[MeSH Terms] OR ("factor"[All Fields] AND "Xa"[All Fields] AND "inhibitors"[All Fields]) OR "factor xa inhibitors"[All Fields] OR ("factor"[All Fields] AND "Xa"[All Fields] AND "inhibitor"[All Fields]) OR "factor xa inhibitor"[All Fields]) OR "factor xa inhibitors"[MeSH Terms] OR "dabigatran"[Supplementary Concept] OR "dabigatran"[MeSH Terms] OR ("dabigatran"[MeSH Terms] OR "dabigatran"[All Fields] OR "dabigatran s"[All Fields]) OR ("rivaroxaban"[MeSH Terms] OR "rivaroxaban"[All Fields]) OR "rivaroxaban"[MeSH Terms] OR "rivaroxaban"[Supplementary Concept] OR "apixaban"[Supplementary Concept] OR ("apixaban"[Supplementary Concept] OR "apixaban"[All Fields] OR "apixaban s"[All Fields]) OR ("edoxaban"[Supplementary Concept] OR "edoxaban"[All Fields]) OR "edoxaban"[Supplementary Concept]) AND ("haemodialysis"[All Fields] OR "renal dialysis"[MeSH Terms] OR ("renal"[All Fields] AND "dialysis"[All Fields]) OR "renal dialysis"[All Fields] OR "hemodialysis"[All Fields])) OR "renal dialysis"[MeSH Terms]) AND ((clinicaltrial[Filter] OR meta-analysis[Filter] OR randomizedcontrolledtrial[Filter]) AND (humans[Filter]) AND (alladult[Filter]))

**Translations**

atrial fibrillation[MeSH Terms]: "atrial fibrillation"[MeSH Terms]

atrial fibrillation: "atrial fibrillation"[MeSH Terms] OR ("atrial"[All Fields] AND "fibrillation"[All Fields]) OR "atrial fibrillation"[All Fields]

atrial fibrillations[MeSH Terms]: "atrial fibrillation"[MeSH Terms]

atrial fibrillations: "atrial fibrillation"[MeSH Terms] OR ("atrial"[All Fields] AND "fibrillation"[All Fields]) OR "atrial fibrillation"[All Fields] OR ("atrial"[All Fields] AND "fibrillations"[All Fields]) OR "atrial fibrillations"[All Fields]

oral: "mouth"[MeSH Terms] OR "mouth"[All Fields] OR "oral"[All Fields]

mouth: "mouth"[MeSH Terms] OR "mouth"[All Fields] OR "mouths"[All Fields] OR "mouth's"[All Fields] OR "mouthed"[All Fields] OR "mouthful"[All Fields] OR "mouthfuls"[All Fields] OR "mouthing"[All Fields]

mouth[MeSH Terms]: "mouth"[MeSH Terms]

anticoagulants[MeSH Terms]: "anticoagulants"[MeSH Terms]

anticoagulants: "anticoagulants"[Pharmacological Action] OR "anticoagulants"[MeSH Terms] OR "anticoagulants"[All Fields] OR "anticoagulant"[All Fields] OR "anticoagulate"[All Fields] OR "anticoagulated"[All Fields] OR "anticoagulating"[All Fields] OR "anticoagulation"[All Fields] OR "anticoagulations"[All Fields] OR "anticoagulative"[All Fields]

NOAC: "N(4)-oleylcytosine arabinoside"[Supplementary Concept] OR "N(4)-oleylcytosine arabinoside"[All Fields] OR "noac"[All Fields]

factor Xa inhibitor: "factor xa inhibitors"[Pharmacological Action] OR "factor xa inhibitors"[MeSH Terms] OR ("factor"[All Fields] AND "xa"[All Fields] AND "inhibitors"[All Fields]) OR "factor xa inhibitors"[All Fields] OR ("factor"[All Fields] AND "xa"[All Fields] AND "inhibitor"[All Fields]) OR "factor xa inhibitor"[All Fields]

factor Xa inhibitor[MeSH Terms]: "factor xa inhibitors"[MeSH Terms]

dabigatran[MeSH Terms]: "dabigatran"[MeSH Terms]

dabigatran: "dabigatran"[MeSH Terms] OR "dabigatran"[All Fields] OR "dabigatran's"[All Fields]

rivaroxaban: "rivaroxaban"[MeSH Terms] OR "rivaroxaban"[All Fields] OR "rivaroxaban's"[All Fields]

rivaroxaban[MeSH Terms]: "rivaroxaban"[MeSH Terms]

apixaban[Supplementary Concept]: "apixaban"[Supplementary Concept]

apixaban: "apixaban"[Supplementary Concept] OR "apixaban"[All Fields] OR "apixaban's"[All Fields]

edoxaban: "edoxaban"[Supplementary Concept] OR "edoxaban"[All Fields] OR "edoxaban's"[All Fields]

edoxaban[Supplementary Concept]: "edoxaban"[Supplementary Concept]

Hemodialysis: "haemodialysis"[All Fields] OR "renal dialysis"[MeSH Terms] OR ("renal"[All Fields] AND "dialysis"[All Fields]) OR "renal dialysis"[All Fields] OR "hemodialysis"[All Fields]

Hemodialysis[MeSH Terms]: "renal dialysis"[MeSH Terms]
