# Supplementary material for: Direct Oral Anticoagulants vs. Warfarin in Hemodialysis Patients With Atrial Fibrillation: A Systematic Review and Meta-Analysis
Source: Front Cardiovasc Med. 2022 Jun 9;9:847286. doi: 10.3389/fcvm.2022.847286 (PMC9218480; doi:10.3389/fcvm.2022.847286)
Supplement: Supplementary file 2 [file Data_Sheet_2.docx]

**Appendix 2: Duration of follow up for each study**

| Author name and study date | Study design | Treatment group (number of patients) | Follow up period in days |
| --- | --- | --- | --- |
| Pokorney et al., 2019 (9) | RCT | Apixaban (82) | 350.5 (211.0 - 458.0) |
|  |  | Warfarin (72) | 350.5 (211.0 - 458.0) |
| Siontis et al., 2018 (10) | retrospective cohort study | Apixaban (2351) | 106-168 |
|  |  | Warfarin (23172) | 175 |
| Chan et al., 2015 (11) | retrospective cohort study | Rivaroxaban (244) | N/A |
|  |  | Warfarin (8064) | N/A |
|  |  | Dabigatran (281) | N/A |
| Sarratt et al., 2017 (12) | retrospective, cohort study | Apixaban (40) | N/A |
|  |  | Warfarin (120) | N/A |
| De Vriese et al. 2020 (13) | RCT | Rivaroxaban (46) | 540 |
|  |  | Warfarin (44) | 540 |

Abbreviations: SD, Standard deviation , N/A not available
